# Experiences of women in secure care who have been prescribed clozapine for borderline personality disorder

**DOI:** 10.1186/s40479-016-0049-x

**Published:** 2016-10-07

**Authors:** Geoffrey L. Dickens, Catherine Frogley, Fiona Mason, Katina Anagnostakis, Marco M. Picchioni

**Affiliations:** 1 Division of Mental Health Nursing and Counselling, School of Social and Health Sciences, Abertay University, Bell Street, Dundee, DD1 1HG UK; 2 St Andrew’s Academic Centre, Northampton, NN1 5DG UK; 3 School of Psychology, Faculty of Arts and Human Sciences, AD Building, University of Surrey, Guildford, Surrey GU2 7XH UK; 4 Medical School, University of Buckingham, Yeomanry House, Hunter Street, Buckingham, MK18 1EG UK; 5 Department of Forensic and Neurodevelopmental Science, King’s College London Institute of Psychiatry Psychology and Neuroscience, De Crespigny Park, Denmark Hill London, SE5 8AF UK

**Keywords:** Clozapine, Borderline personality disorder, Emotionally unstable personality disorder, Thematic analysis

## Abstract

**Background:**

Clozapine is an atypical antipsychotic medicine which can cause significant side-effects. It is often prescribed off-license in severe cases of borderline personality disorder contrary to national treatment guidelines. Little is known about the experiences of those who take clozapine for borderline personality disorder. We explored the lived-experience of women in secure inpatient care who were prescribed clozapine for borderline personality disorder.

**Findings:**

Adult females (*N* = 20) participated in audio-taped semi-structured interviews. Transcripts were subject to thematic analysis. The central themes related to evaluation, wellbeing, understanding and self-management; for many, their subjective wellbeing on clozapine was preferred to prior levels of functioning and symptomatology, sometimes profoundly so. The negative and potentially adverse effects of clozapine were explained as regrettable but relatively unimportant.

**Conclusions:**

When psychological interventions are, at least initially, ineffective then clozapine treatment is likely to be evaluated positively by a group of women with borderline personality disorder in secure care despite the potential disadvantages.

## Background

People with borderline personality disorder (BPD) experience pervasive instability of affect, self-image, impulse control, behaviour, and relationships [1]. Self-harm, use of emergency health services, and impulsive aggression are common [2–5]. Guidelines recommend psychosocial interventions [6–8], limiting the role of medicines to crises or psychiatric co-morbidity. Nevertheless, clozapine is commonly prescribed for severely disturbed BPD patients [9], a controversial practice both because of its significant side-effect profile, including potentially fatal severe neutropenia, and an absence of supporting randomised controlled trials (RCTs). Two non-RCT studies found psychotic and depressive symptom-related and global functioning improvements among a subset of BPD-patients with prolonged and/or pronounced psychotic symptoms [10, 11]. One further study, our own recent retrospective case-series [12], suggested a number of objective benefits for women diagnosed with BPD displaying severe symptoms and resident in a secure mental health service. Despite this, there are currently no published studies of patients’ personal subjective experiences of taking clozapine for BPD. In this study we conducted semi-structured interviews with the same women participants to explore their personal experience of clozapine.

## Methods

Inclusion criteria: women aged 18 to 65 diagnosed with BPD and treated with clozapine while inpatients at St Andrew’s Healthcare between 2002 and 2010. Exclusion criteria: learning disability, diagnosis of schizophrenia or other psychotic disorder. Consenting participants were interviewed for up to 1 h. The interview comprised items about clozapine initiation, and their experience of taking it. Emerging issues were explored through unscripted prompts and cues. Interviews were audio-recorded, transcribed and subjected to a six stage thematic analysis [13]: i) transcripts were read repeatedly; ii) independent open coding of narrative data; iii) codes were compared, discussed, and, where necessary, amalgamated or split; iv) segments with similar codes were mapped; v) segments were linked hierarchically into sub-themes and combined into themes; vi) further recoding following review and discussion by the research team to ensure congruence between the presented extracts and each theme. Our guiding approach was essentialist-realist since we assumed that what participants told us reflected the reality as they saw it.

## Results

Thirty women were eligible and 20 agreed to participate (response rate 67 %). Mean age of participants at clozapine initiation was 27.8 years (SD = 7.8); mean time on clozapine was 26.5 months (SD = 23.0); 15 (75.0 %) patients were detained under forensic commitment at initiation and 5 (25.0 %) under civil commitment; almost all (*n* = 18, 90.0 %) had a history of previous admissions to secure services; all had received other antipsychotics prior to clozapine (Mode = 3; range 1 to 6). Analysis revealed four themes.

### Evaluation

Participants described an active personal evaluation of clozapine’s effects, expressed as a perceived benefit-loss trade-off. Benefits seemed profound but were not always articulated precisely:

*“It’s just hard to explain the difference, because a lot of people have noticed the difference with me since I’ve been on it* [clozapine]*, but it’s hard to explain what it feels like inside”* (P15)

*“I wish it had been around years ago. Literally, you know. It’s been really, really good, it’s been a really positive experience.”* (P8)

*“… it closed my emotions off. It closed me off… I literally closed down. I can’t feel anything. I’m more like a robot not a woman.”* (P20)

Most women mentioned significant side-effects, notably hypersalivation (*n* = 11 at 6 month follow-up). In addition, there was a mean weight gain of 7.8 Kg at 6 month follow-up. However, side effect importance appeared comparatively low within an overall positive evaluation of clozapine.

*“In the beginning I was very tired… but I’ve managed to pull myself out of that… which comes to the second point, which is becoming overweight. But I have had weight problems before I went on to clozapine, so it’s not solely dependent on clozapine. The clozapine hasn’t helped my weight at all. But I think it, erm, if it’s managed properly, I think you can control your weight with clozapine, you know, maybe some exercise”* (P8)

*“The weight gain well I deal with that my way, that’s just an ongoing thing. I do things about that so it’s alright; it’s not the end of the world is it?”* (P20)

Some participants drew positive comparisons between clozapine and other antipsychotics they had experienced, all in clozapine’s favour.

*“I was taking 40* [mg] *olanzapine a day… I was still struggling, I was still crying, still doing risk behaviours and everything. Since I got put on clozapine I’ve built it up slowly and I haven’t done anything since. I’ve tried loads of drugs, loads and loads of different ones but clozapine is best out of all of them”* (P18)

### Wellbeing

Almost all (19/20) participants reported tangible benefits from taking prescribed clozapine including cognitive and affective changes:

*“I’m not in turmoil anymore. I just see it as what will be will be and I am who I am…I deal with things a lot better, it doesn’t raise so many issues… I suppose my impulse control has gone down, my impulses have gone down”* (P16)

*“I used to be like… climbing up the ceiling and then dropping really flat. That hasn’t happened since the clozapine”* (P19)

Spontaneous comments of behavioural and social improvement were made by a substantial proportion of participants including reduced impulsivity and aggression/self-harm, and improved relationships. Where clozapine had commenced then stopped participants noted that the gains made were subsequently lost. One woman said that clozapine had negatively affected her impulsivity and interpersonal relationships. Some described becoming ‘normal’ while others described something more profound:

*“I’ve always called it the magic pill because other medications have made me better…but they masked my illness. But with clozapine it’s not that it makes me better, it completely turns me into a normal person. It’s not masking something it takes it away”* (P2)

*“it’s given me freedom of thought, speech…it’s made me well…made me happy. It’s made me someone that I’ve never been able to be before. I couldn’t hold a conversation like this years ago, I would have just been rude, obnoxious, scared…paranoid, self-harming every day, just like a shell of a person”* (P6)

### Understanding

This theme encompassed women’s expressed understanding of BPD within a medical framework:

*“Although we get the depressive thoughts, it isn’t actually clinical depression so for Borderlines* [sic] *it* [anti-depressant medication] *doesn’t work. So yeah, all the other medication I’ve been on obviously hasn’t helped me but in the last 18 months clozapine has really helped me”* (P7)

Participant 3 understood her behaviour, which she described as “aggressive and lashing out” prior to clozapine initiation in illness-defined terms:

*“Because I was really poorly. I had really poor behaviour, but not just because I wanted to be, but because I couldn’t be anything else”*

Some also understood the effects of clozapine through observation of fellow patients:

*“I asked to be put on it. Because I saw what it was like when the other girls here took it. I just said I wanted to go on it because it helps the other girls”* (P5)

Participants sometimes came to new understanding of the change attributed to clozapine from feedback from others:

*“One poor nurse, I don’t remember but I was told afterwards, she said she was scared of me. You don’t realise how you were before you are put on clozapine”* (P9)

### Self-management

Central to self-management was the relationship with the prescribing psychiatrist who had recommended the medicine.

*“We sat down and they talked to me about my illness, which is Post Traumatic Stress Disorder and Borderline Personality Disorder, and that this medication worked really well in people with my condition, and that they had been using it for a while and that it’s been really good and they wanted to try me on it”* (P2)

For some clozapine was viewed as the primary method by which they would continue to manage their BPD, while some expressed that clozapine facilitated other aspects of their care and treatment:

*“I wouldn’t ever stop taking it, put it that way. It’s annoying but I would never stop taking it”* (P19)

*“I’m really stable and I’ve started applying the skills that we learn in DBT. If at times I get upset about anything or whatever, I can quickly bring myself back to, you know, dealing with things appropriately, rather than going completely off the rails… And I feel like I can feel the emotions that I feel but with the clozapine it helps me to do it in a more balanced way”* (P14)

## Discussion

The largely positive results are consistent with our recent study [10] and other non-RCT evidence [10, 11, 14]. They challenge a universal pharmacology-free BPD-recovery paradigm. Nevertheless, the congruence was unexpected since clozapine’s side-effect profile is so marked. Even serious disadvantages were described matter-of-factly, and the risks considered tolerable. Only psychosocial approaches are recommended for BPD. However, some people experience Dialectical Behaviour Therapy as too focused on self-harm, and less helpful for self-confidence, emotions and relatonships [15], the areas that women in this study reported the most progress on. In the absence of an RCT, many practitioners already prescribe clozapine for BPD off-licence [9]. Their practice is increasingly evidence-based and, most importantly, valued by many patients. The results are also consistent with data that non-BPD patients prescribed antipsychotics are quick and decisive in their judgement of them as ‘good’ or ‘bad’ [16]. To reduce reporting bias, interviews were conducted by an assistant psychologist who had no clinical role with the participants. The interview style encouraged reporting of all experiences, while confidentiality and anonymity were assured. We invited all eligible patients who had received clozapine for BPD to take part. It is possible that the one third who declined were less satisfied, though that does not negate the experiences of the other participants. The sample included many detained women with very complex needs. Clinicians should communicate clearly with BPD-diagnosed patients about clozapine treatment. People with BPD seem to be generally dissatisfied with medication-oriented approaches [16]. However, it is premature to generalise findings from medicines in general to clozapine in particular. Participants were sanguine about their experience: this suggests that good communication with BPD patients around the reasons for prescribing clozapine is essential. Development in the US of a shared risk evaluation and mitigation strategy (REMS) called the Clozapine REMS Program which requires central monitoring of prescribing, dispensing, and receipt of clozapine should further increase confidence in the safety of the drug which may assist in aiding balanced decision+s about. In the absence of gold standard evidence this study and our related study [12] suggests that therapeutic nihilism about this group should be challenged: severe cases of BPD can be treated successfully, women value the treatment, and tolerate inconvenience and side effects because the gains can be profound.

Finally, we cannot overstress the importance of the need for well-designed RCTs to rigorously test the efficacy of clozapineon all sub-syndromal symptoms of the disorder in people with moderate to severe BPD.

## Abbreviations

BPD: Borderline personality disorder
RCT: Randomised controlled trial
